# Supplementary material for: Surveillance of Amphotericin B and Azole Resistance in Aspergillus Isolated from Patients in a Tertiary Teaching Hospital
Source: J Fungi (Basel). 2023 Nov 1;9(11):1070. doi: 10.3390/jof9111070 (PMC10672583; doi:10.3390/jof9111070)
Supplement: Supplementary file 1 [file jof-09-01070-s001.zip › Supplementary Table S1.docx]

SUPPLEMENTARY MATERIAL

Supplementary Table S1 – Molecular identification of *Aspergillus* spp. clinical isolates. ITS, internal transcribed spacer of ribossomal DNA; *CaM*, calmodulin coding gene.

| **Clinical Isolate ID** | **ITS** | | | | | ***CaM*** | | | | |
| --- | --- | --- | --- | --- | --- | --- | --- | --- | --- | --- |
|  | **Species** | **GenBank Ref. Seq.** | **Identity (%)** | **Máx. Score** | **E- value** | **Species** | **GenBank Ref. Seq.** | **Identity (%)** | **Máx. Score** | **E- value** |
| LMC6007.01 | *A. parasiticus* | AY373859 | 99.57 | 852 | 0.0 | *A. parasiticus* | AY017584 | 99.8 | 926 | 0.0 |
| LMC6008.01 | *A. parasiticus* | AY373859 | 99.57 | 852 | 0.0 | *A. parasiticus* | AY017584 | 99.8 | 926 | 0.0 |
| LMC6009.01 | *A. parasiticus* | AY373859 | 99.57 | 852 | 0.0 | *A. parasiticus* | AY017584 | 99.8 | 926 | 0.0 |
| LMC6010.01 | *A. fumigatus* | AY373859 | 100 | 876 | 0.0 | *A. fumigatus* | EF669860 | 100 | 861 | 0.0 |
| LMC6010.02 | *A. parasiticus* | AY373859 | 99.57 | 852 | 0.0 | *A. parasiticus* | AY017584 | 99.8 | 926 | 0.0 |
| LMC6011.01 | *A. fumigatus* | EF669931 | 100 | 876 | 0.0 | *A. fumigatus* | EF669860 | 100 | 861 | 0.0 |
| LMC6012.01 | *A. parasiticus* | AY373859 | 99.57 | 852 | 0.0 | *A. parasiticus* | AY017584 | 99.8 | 926 | 0.0 |
| LMC6013.01 | *A. fumigatus* | EF669931 | 100 | 876 | 0.0 | *A. fumigatus* | EF669860 | 100 | 861 | 0.0 |
| LMC6014.01 | *A. fumigatus* | EF669931 | 100 | 876 | 0.0 | *A. fumigatus* | EF669860 | 100 | 861 | 0.0 |
| LMC6015.01 | *A. fumigatus* | EF669931 | 100 | 876 | 0.0 | *A. fumigatus* | EF669860 | 100 | 861 | 0.0 |
| LMC6016.01 | *A. fumigatus* | EF669931 | 100 | 876 | 0.0 | *A. fumigatus* | EF669860 | 100 | 861 | 0.0 |
| LMC6017.02 | *A. fumigatus* | EF669931 | 100 | 876 | 0.0 | *A. fumigatus* | EF669860 | 100 | 861 | 0.0 |
| LMC6017.03 | *A. fumigatus* | EF669931 | 100 | 876 | 0.0 | *A. fumigatus* | EF669860 | 100 | 861 | 0.0 |
| LMC6018.01 | *A. fumigatus* | EF669931 | 100 | 876 | 0.0 | *A. fumigatus* | EF669860 | 100 | 861 | 0.0 |
| LMC6019.01 | *A. fumigatus* | EF669931 | 100 | 876 | 0.0 | *A. fumigatus* | EF669860 | 100 | 861 | 0.0 |
| LMC6020.01 | *A. fumigatus* | EF669931 | 100 | 876 | 0.0 | *A. fumigatus* | EF669860 | 100 | 861 | 0.0 |
| LMC6021.01 | *A. parasiticus* | AY373859 | 99.57 | 852 | 0.0 | *A. parasiticus* | AY017584 | 99.8 | 926 | 0.0 |
| LMC6022.01 | *A. flavus* | AF027863 | 100 | 869 | 0.0 | *A. flavus* | EF661508 | 99.4 | 942 | 0.0 |
| LMC6023.01 | *A. tamarii* | AF004929 | 98.7 | 863 | 0.0 | *A. tamarii* | EF661526 | 99.8 | 926 | 0.0 |
| LMC6023.02 | *A. fumigatus* | EF669931 | 100 | 876 | 0.0 | *A. fumigatus* | EF669860 | 100 | 861 | 0.0 |
| LMC6023.03 | *A. parasiticus* | AY373859 | 99.57 | 852 | 0.0 | *A. parasiticus* | AY017584 | 99.8 | 926 | 0.0 |
| LMC6024.01 | *A. flavus* | AF027863 | 100 | 869 | 0.0 | *A. flavus* | EF661508 | 99.4 | 942 | 0.0 |
| LMC6025.01 | *A. fumigatus* | EF669931 | 100 | 876 | 0.0 | *A. fumigatus* | EF669860 | 100 | 861 | 0.0 |
| LMC6026.01 | *A. flavus* | AF027863 | 100 | 869 | 0.0 | *A. flavus* | EF661508 | 99.4 | 942 | 0.0 |
| LMC6027.01 | *A. tamarii* | AF004929 | 98.5 | 869 | 0.0 | *A. tamarii* | EF661526 | 99.8 | 926 | 0.0 |
| LMC6028.01 | *A. parasiticus* | AY373859 | 99.57 | 852 | 0.0 | *A. parasiticus* | AY017584 | 99.8 | 926 | 0.0 |
| LMC6029.01 | *A. parasiticus* | AY373859 | 99.57 | 852 | 0.0 | *A. parasiticus* | AY017584 | 99.8 | 926 | 0.0 |
| LMC6030.01 | *A. flavus* | AF027863 | 100 | 869 | 0.0 | *A. flavus* | EF661508 | 99.4 | 942 | 0.0 |
| LMC6031.01 | *A. flavus* | AF027863 | 100 | 869 | 0.0 | *A. flavus* | EF661508 | 99.4 | 942 | 0.0 |
| LMC8001.01 | *A. fumigatus* | EF669931 | 100 | 876 | 0.0 | *A. fumigatus* | EF669860 | 100 | 861 | 0.0 |
| LMC8001.03 | *A. fumigatus* | EF669931 | 100 | 876 | 0.0 | *A. fumigatus* | EF669860 | 100 | 861 | 0.0 |
| LMC8001.05 | *A. fumigatus* | EF669931 | 100 | 876 | 0.0 | *A. fumigatus* | EF669860 | 100 | 861 | 0.0 |
| LMC8001.06 | *A. fumigatus* | EF669931 | 100 | 876 | 0.0 | *A. fumigatus* | EF669860 | 100 | 861 | 0.0 |
| LMC8003.01 | *A. fumigatus* | EF669931 | 100 | 876 | 0.0 | *A. fumigatus* | EF669860 | 100 | 861 | 0.0 |
| LMC8003.02 | *A. fumigatus* | EF669931 | 100 | 876 | 0.0 | *A. fumigatus* | EF669860 | 100 | 861 | 0.0 |
| LMC8003.05 | *A. fumigatus* | EF669931 | 100 | 876 | 0.0 | *A. fumigatus* | EF669860 | 100 | 861 | 0.0 |
| LMC8003.06 | *A. fumigatus* | EF669931 | 100 | 876 | 0.0 | *A. fumigatus* | EF669860 | 100 | 861 | 0.0 |
| LMC8003.13 | *A. fumigatus* | EF669931 | 100 | 876 | 0.0 | *A. fumigatus* | EF669860 | 100 | 861 | 0.0 |
| LMC9001.01 | *A. flavus* | AF027863 | 100 | 869 | 0.0 | *A. flavus* | EF661486* | 99.5 | 780 | 0.0 |
| LMC9002.01 | *A. flavus* | AF027863 | 100 | 869 | 0.0 | *A. flavus* | EF661486* | 99.8 | 883 | 0.0 |
| LMC9003.01 | *A. fumigatus* | EF669931 | 100 | 876 | 0.0 | *A. fumigatus* | EF669860 | 100 | 861 | 0.0 |
| LMC9004.01 | *A. fumigatus* | EF669931 | 100 | 876 | 0.0 | *A. fumigatus* | EF669860 | 100 | 861 | 0.0 |
| LMC9005.01 | *A. flavus* | AF027863 | 100 | 869 | 0.0 | *A. flavus* | EF661486* | 99.8 | 883 | 0.0 |
| LMC9006.01 | *A. flavus* | AF027863 | 100 | 869 | 0.0 | *A. flavus* | EF661486* | 99.5 | 780 | 0.0 |
| LMC9007.01 | *A. flavus* | AF027863 | 100 | 869 | 0.0 | *A. flavus* | EF661486* | 99.8 | 883 | 0.0 |
| LMC9008.01 | *A. fumigatus* | EF669931 | 100 | 876 | 0.0 | *A. fumigatus* | EF669860 | 100 | 861 | 0.0 |
| LMC9009.01 | *A. fumigatus* | EF669931 | 100 | 876 | 0.0 | *A. fumigatus* | EF669860 | 99.55 | 809 | 0.0 |
| LMC9010.01 | *A. flavus* | AF027863 | 100 | 869 | 0.0 | *A. flavus* | EF661486* | 98.5 | 850 | 0.0 |
| LMC9011.01 | *A. flavus* | AF027863 | 100 | 869 | 0.0 | *A. flavus* | EF661486* | 99.8 | 883 | 0.0 |
| LMC9012.01 | *A. flavus* | AF027863 | 100 | 869 | 0.0 | *A. flavus* | EF661486* | 99.8 | 883 | 0.0 |
| LMC9013.01 | *A. fumigatus* | EF669931 | 100 | 876 | 0.0 | *A. fumigatus* | EF669860 | 99.8 | 856 | 0.0 |
| LMC9014.01 | *A. fumigatus* | EF669931 | 100 | 876 | 0.0 | *A. fumigatus* | EF669860 | 99.5 | 869 | 0.0 |
| LMC9015.01 | *A. fumigatus* | EF669931 | 100 | 876 | 0.0 | *A. fumigatus* | EF669860 | 100 | 848 | 0.0 |
| LMC9016.01 | *A. fumigatus* | EF669931 | 100 | 876 | 0.0 | *A. fumigatus* | EF669860 | 100 | 841 | 0.0 |
| LMC9017.01 | *A. fumigatus* | EF669931 | 100 | 876 | 0.0 | *A. fumigatus* | EF669860 | 99.1 | 830 | 0.0 |
| LMC9018.01 | *A. fumigatus* | EF669931 | 100 | 876 | 0.0 | *A. fumigatus* | EF669860 | 100 | 861 | 0.0 |
| LMC9019.01 | *A. fumigatus* | EF669931 | 100 | 876 | 0.0 | *A. fumigatus* | EF669860 | 98.86 | 785 | 0.0 |
| LMC9020.01 | *A. fumigatus* | EF669931 | 100 | 876 | 0.0 | *A. fumigatus* | EF669860 | 100 | 845 | 0.0 |
| LMC9021.01 | *A. fumigatus* | EF669931 | 100 | 876 | 0.0 | *A. fumigatus* | EF669860 | 100 | 837 | 0.0 |
| LMC9022.01 | *A. fumigatus* | EF669931 | 100 | 876 | 0.0 | *A. fumigatus* | EF669860 | 100 | 861 | 0.0 |
| LMC9023.01 | *A. fumigatus* | EF669931 | 100 | 876 | 0.0 | *A. fumigatus* | EF669860 | 99.8 | 856 | 0.0 |
| LMC9024.01 | *A. fumigatus* | EF669931 | 100 | 876 | 0.0 | *A. fumigatus* | EF669860 | 100 | 861 | 0.0 |
| LMC9025.01 | *A. fumigatus* | EF669931 | 100 | 876 | 0.0 | *A. fumigatus* | EF669860 | 99.8 | 835 | 0.0 |
| LMC9026.01 | *A. fumigatus* | EF669931 | 100 | 876 | 0.0 | *A. fumigatus* | EF669860 | 100 | 861 | 0.0 |
| *, *benA* sequencing | | | | | | | | | | |
