# Supplementary material for: Surveillance of Amphotericin B and Azole Resistance in Aspergillus Isolated from Patients in a Tertiary Teaching Hospital
Source: J Fungi (Basel). 2023 Nov 1;9(11):1070. doi: 10.3390/jof9111070 (PMC10672583; doi:10.3390/jof9111070)
Supplement: Supplementary file 1 [file jof-09-01070-s001.zip › Supplementary Table S2.docx]

SUPPLEMENTARY MATERIAL

Table S2 – *Aspergillus* spp. clinical isolates Genbank accession numbers.

| **Clinical Isolate ID** | **Species** | **Genbank Accession number** | | |
| --- | --- | --- | --- | --- |
|  |  | **ITS** | ***CaM*** | ***cyp51A*** |
| LMC6007.01 | *A. parasiticus* | OR095646 | OR100829 | − |
| LMC6008.01 | *A. parasiticus* | OR095647 | OR100830 | − |
| LMC6009.01 | *A. parasiticus* | OR095648 | OR100831 | − |
| LMC6010.01 | *A. fumigatus s.s.* | OR095583 | OR100789 | OR100868 |
| LMC6010.02 | *A. parasiticus* | OR095649 | OR100832 | − |
| LMC6011.01 | *A. fumigatus s.s.* | OR095584 | OR100790 | OR100869 |
| LMC6012.01 | *A. parasiticus* | OR095650 | OR100833 | − |
| LMC6013.01 | *A. fumigatus s.s.* | OR095585 | OR100791 | OR100870 |
| LMC6014.01 | *A. fumigatus s.s.* | OR095586 | OR100792 | OR100871 |
| LMC6015.01 | *A. fumigatus s.s.* | OR095587 | OR100793 | OR100872 |
| LMC6016.01 | *A. fumigatus s.s.* | OR095588 | OR100794 | OR100873 |
| LMC6017.02 | *A. fumigatus s.s.* | OR095589 | OR100795 | OR100874 |
| LMC6017.03 | *A. fumigatus s.s.* | OR095590 | OR100796 | OR100875 |
| LMC6018.01 | *A. fumigatus s.s.* | OR095591 | OR100797 | OR100876 |
| LMC6019.01 | *A. fumigatus s.s.* | OR095592 | OR100798 | OR100877 |
| LMC6020.01 | *A. fumigatus s.s.* | OR095593 | OR100799 | OR100878 |
| LMC6021.01 | *A. parasiticus* | OR095651 | OR100834 | − |
| LMC6022.01 | *A. flavus* | OR095496 | OR100784 | − |
| LMC6023.01 | *A. tamarii* | OR095644 | OR100838 | − |
| LMC6023.02 | *A. fumigatus s.s.* | OR095594 | OR100800 | OR100879 |
| LMC6023.03 | *A. parasiticus* | OR095652 | OR100835 | − |
| LMC6024.01 | *A. flavus* | OR095497 | OR100785 | − |
| LMC6025.01 | *A. fumigatus s.s.* | OR095595 | OR100801 | OR100880 |
| LMC6026.01 | *A. flavus* | OR095498 | OR100786 | − |
| LMC6027.01 | *A. tamarii* | OR095645 | OR100839 | − |
| LMC6028.01 | *A. parasiticus* | OR095653 | OR100836 | − |
| LMC6029.01 | *A. parasiticus* | OR095654 | OR100837 | − |
| LMC6030.01 | *A. flavus* | OR095499 | OR100787 | − |
| LMC6031.01 | *A. flavus* | OR095500 | OR100788 | − |
| LMC8001.01 | *A. fumigatus s.s.* | OR095596 | OR100802 | OR100840 |
| LMC8001.03 | *A. fumigatus s.s.* | OR095597 | OR100803 | OR100841 |
| LMC8001.05 | *A. fumigatus s.s.* | OR095598 | OR100804 | OR100842 |
| LMC8001.06 | *A. fumigatus s.s.* | OR095599 | OR100805 | OR100843 |
| LMC8003.01 | *A. fumigatus s.s.* | OR095600 | OR100806 | OR100844 |
| LMC8003.02 | *A. fumigatus s.s.* | OR095601 | OR100807 | OR100845 |
| LMC8003.05 | *A. fumigatus s.s.* | OR095602 | OR100808 | OR100847 |
| LMC8003.06 | *A. fumigatus s.s.* | OR095603 | OR100809 | OR100848 |
| LMC8003.13 | *A. fumigatus s.s.* | OR095604 | OR100810 | OR100849 |
| LMC9001.01 | *A. flavus* | OR095501 | OR225627* | − |
| LMC9002.01 | *A. flavus* | OR095502 | OR225628* | − |
| LMC9003.01 | *A. fumigatus s.s.* | OR095605 | OR100811 | OR100850 |
| LMC9004.01 | *A. fumigatus s.s.* | OR095606 | OR100812 | OR100851 |
| LMC9005.01 | *A. flavus* | OR095503 | OR225629* | − |
| LMC9006.01 | *A. flavus* | OR095504 | OR225630* | − |
| LMC9007.01 | *A. flavus* | OR095505 | OR225631* | − |
| LMC9008.01 | *A. fumigatus s.s.* | OR095607 | OR100813 | OR100852 |
| LMC9009.01 | *A. fumigatus s.s.* | OR095608 | OR100814 | OR100853 |
| LMC9010.01 | *A. flavus* | OR095506 | OR225632* | − |
| LMC9011.01 | *A. flavus* | OR095507 | OR225633* | − |
| LMC9012.01 | *A. flavus* | OR095508 | OR225634* | − |
| LMC9013.01 | *A. fumigatus s.s.* | OR095609 | OR100815 | OR100854 |
| LMC9014.01 | *A. fumigatus s.s.* | OR095610 | OR100816 | OR100855 |
| LMC9015.01 | *A. fumigatus s.s.* | OR095611 | OR100817 | OR100856 |
| LMC9016.01 | *A. fumigatus s.s.* | OR095612 | OR100818 | OR100857 |
| LMC9017.01 | *A. fumigatus s.s.* | OR095613 | OR100819 | OR100858 |
| LMC9018.01 | *A. fumigatus s.s.* | OR095614 | OR100820 | OR100859 |
| LMC9019.01 | *A. fumigatus s.s.* | OR095615 | OR100821 | OR100860 |
| LMC9020.01 | *A. fumigatus s.s.* | OR095616 | OR100822 | OR100861 |
| LMC9021.01 | *A. fumigatus s.s.* | OR095617 | OR100823 | OR100862 |
| LMC9022.01 | *A. fumigatus s.s.* | OR095618 | OR100824 | OR100863 |
| LMC9023.01 | *A. fumigatus s.s.* | OR095619 | OR100825 | OR100864 |
| LMC9024.01 | *A. fumigatus s.s.* | OR095620 | OR100826 | OR100865 |
| LMC9025.01 | *A. fumigatus s.s.* | OR095621 | OR100827 | OR100866 |
| LMC9026.01 | *A. fumigatus s.s.* | OR095622 | OR100828 | OR100867 |
| ITS, internal trabscribed spacer of ribosomal DNA; *CaM*, calmodulin coding gene; *, *benA* (ß-tubulin coding gene) sequencing. | | | | |
