# Supplementary material for: Surveillance of Amphotericin B and Azole Resistance in Aspergillus Isolated from Patients in a Tertiary Teaching Hospital
Source: J Fungi (Basel). 2023 Nov 1;9(11):1070. doi: 10.3390/jof9111070 (PMC10672583; doi:10.3390/jof9111070)
Supplement: Supplementary file 1 [file jof-09-01070-s001.zip › Supplementary Table S3.docx]

Supplementary Table S3 – *Aspergillus* spp. clinical isolates and general results of the study.

| **Clinical Isolate ID** | **Species** | **Cyp51A aminoacids substitutions** | | | | | | **MIC (µg/mL)** | | | | **Biofilm** | **Virulence** |
| --- | --- | --- | --- | --- | --- | --- | --- | --- | --- | --- | --- | --- | --- |
|  |  | **F46Y** | **M172V** | **N248T** | **N248K** | **D255E** | **E427K** | **ITR** | **POS** | **VOR** | **AMB** |  |  |
| LMC6007.01 | *A. parasiticus* | NP |  |  |  |  |  | 0.5 | 1* | 1 | 2 | + | +++ |
| LMC6008.01 | *A. parasiticus* | NP |  |  |  |  |  | 0.5 | 0.5 | 4* | 2 | + | +++ |
| LMC6009.01 | *A. parasiticus* | NP |  |  |  |  |  | 0.5 | 0.5 | 0.5 | 2 | ++ | +++ |
| LMC6010.01^§ c^ | *A. fumigatus s.s.* | - | M172V | - | - | - | - | 0.5 | 0.5* | 2* | 0.5 | ++ | + |
| LMC6010.02 | *A. parasiticus* | NP |  |  |  |  |  | 1 | 0.5 | 2 | 2 | + |  |
| LMC6011.01^§ c^ | *A. fumigatus s.s.* | - | M172V | - | - | - | - | 16* | 8* | 2* | 1 | ++ | + |
| LMC6012.01 | *A. parasiticus* | NP |  |  |  |  |  | 0.5 | 1* | 2 | 2 | ++ | +++ |
| LMC6013.01 | *A. fumigatus s.s.* | - | - | - | - | - | - | 1 | 0.5* | 4* | 1 | ++ | + |
| LMC6014.01 | *A. fumigatus s.s.* | - | - | - | - | - | - | 0.5 | 0.5* | 2* | 1 | ++ | ++ |
| LMC6015.01^§ c^ | *A. fumigatus s.s.* | F46Y | M172V | - | - | - | E427K | 1 | 1* | 4* | 1 | ++ | + |
| LMC6016.01 | *A. fumigatus s.s.* | - | - | - | - | - | - | 0.5 | 1* | 2* | 1 | ++ | + |
| LMC6017.02^§ c^ | *A. fumigatus s.s.* | F46Y | M172V | N248T | - | D255E | E427K | 1 | 0.5* | 2* | 2 | ++ | + |
| LMC6017.03^§ c^ | *A. fumigatus s.s.* | F46Y | M172V | N248T | - | D255E | E427K | 1 | 0.5* | 8* | 2 | ++ | + |
| LMC6018.01^§ c^ | *A. fumigatus s.s.* | F46Y | M172V | N248T | - | D255E | E427K | 2* | 1* | 2* | 1 | ++ | +++ |
| LMC6019.01 | *A. fumigatus s.s.* | - | - | - | - | - | - | 0.5 | 0.5* | 2* | 1 | ++ | ++ |
| LMC6020.01 | *A. fumigatus s.s.* | - | - | - | - | - | - | 0.5 | 0.5* | 1 | 1 | ++ | + |
| LMC6021.01 | *A. parasiticus* | NP |  |  |  |  |  | 1 | 0.5 | 2 | 2 | ++ | +++ |
| LMC6022.01 | *A. flavus* | NP |  |  |  |  |  | 1 | 0.5 | 4* | 1 | ++ | +++ |
| LMC6023.01 | *A. tamarii* | NP |  |  |  |  |  | 1 | 0.06 | 1 | 0.5 | ++ | +++ |
| LMC6023.02^§ c^ | *A. fumigatus s.s.* | - | M172V | - | - | - | - | 1 | 0.5* | 2* | 0.5 | ++ | + |
| LMC6023.03 | *A. parasiticus* | NP |  |  |  |  |  | 1 | 0.5 | 2 | 4 | ++ | +++ |
| LMC6024.01 | *A. flavus* | NP |  |  |  |  |  | 1 | 0.12 | 4* | 1 | ++ | +++ |
| LMC6025.01^§ c^ | *A. fumigatus s.s.* | F46Y | M172V | N248T | - | D255E | E427K | 1 | 0.5* | 2* | 1 | +++ | + |
| LMC6026.01 | *A. flavus* | NP |  |  |  |  |  | 1 | 0.12 | 2 | 1 | ++ | +++ |
| LMC6027.01 | *A. tamarii* | NP |  |  |  |  |  | 1 | 0.03 | 2 | 0.5 | +++ | +++ |
| LMC6028.01 | *A. parasiticus* | NP |  |  |  |  |  | 0.5 | 1* | 2 | 4 | + | +++ |
| LMC6029.01 | *A. parasiticus* | NP |  |  |  |  |  | 1 | 0.12 | 2 | 4 | ++ | +++ |
| LMC6030.01 | *A. flavus* | NP |  |  |  |  |  | 0.5 | 0.5 | 2 | 2 | +++ | +++ |
| LMC6031.01 | *A. flavus* | NP |  |  |  |  |  | 1 | 0.12 | 4* | 2 | ++ | +++ |
| LMC8001.01 | *A. fumigatus s.s.* | - | - | - | - | - | - | 0.5 | 0.06 | 0.25 | 4* | ++ | +++ |
| LMC8001.03 | *A. fumigatus s.s.* | - | - | - | - | - | - | 0.5 | 0.12 | 0.5 | 2 | + | ++ |
| LMC8001.05 | *A. fumigatus s.s.* | - | - | - | - | - | - | 0.5 | 0.12 | 1 | 2 | + |  |
| LMC8001.06 | *A. fumigatus s.s.* | - | - | - | - | - | - | 0.5 | 0.12 | 0.25 | 2 | + |  |
| LMC9025.01 | *A. fumigatus s.s.* | - | - | - | - | - | - | 0.5 | 0.12 | 1 | 2 | ++ |  |
| LMC8003.01 | *A. fumigatus s.s.* | - | - | - | - | - | - | 0.5 | 0.25 | 0.5 | 4* | ++ |  |
| LMC8003.02^§ c^ | *A. fumigatus s.s.* | - | M172V | - | - | - | - | 0.5 | 0.5* | 0.5 | 2 | ++ |  |
| LMC8003.05^§ c^ | *A. fumigatus s.s.* | - | M172V | - | - | - | - | 0.5 | 0.12 | 0.5 | 2 | ++ |  |
| LMC8003.06 | *A. fumigatus s.s.* | - | - | - | - | - | - | 0.5 | <0.03 | 0.5 | 2 | + |  |
| LMC8003.13^§ c^ | *A. fumigatus s.s.* | - | M172V | - | - | - | - | 0.5 | 1* | 0.5 | 2 | + |  |
| LMC9026.01^§ c^ | *A. fumigatus s.s.* | - | M127V | - | - | - | - | 0.5 | 0.5* | 0.25 | 2 | ++ | ++ |
| LMC9001.01 | *A. flavus* | NP |  |  |  |  |  | 0.5 | 0.5 | 0.25 | 2 | ++ | +++ |
| LMC9002.01 | *A. flavus* | NP |  |  |  |  |  | 0.5 | 0.5 | 0.25 | 2 | ++ |  |
| LMC9003.01^§ c^ | *A. fumigatus s.s.* | F46Y | M172V | N248T | - | D255E | E427K | 8* | >16* | 0.5 | >16* | + | + |
| LMC9004.01^§ c^ | *A. fumigatus s.s.* | F46Y | M172V | N248T | - | D255E | E427K | 0.5 | 1* | 0.5 | 2 | + |  |
| LMC9005.01 | *A. flavus* | NP |  |  |  |  |  | 0.25 | 0.5 | 0.25 | 2 | ++ | + |
| LMC9006.01 | *A. flavus* | NP |  |  |  |  |  | 0.25 | 0.5 | 0.5 | 2 | ++ |  |
| LMC9007.01 | *A. flavus* | NP |  |  |  |  |  | 0.25 | 0.5 | 0.25 | 2 | ++ | +++ |
| LMC9008.01^§ c^ | *A. fumigatus s.s.* | F46Y | M172V | N248T | - | D255E | E427K | 0.5 | 1* | 0.5 | 2 | + |  |
| LMC9009.01 | *A. fumigatus s.s.* | - | - | - | - | - | - | 0.5 | 0.5* | 0.5 | 2 | ++ |  |
| LMC9010.01 | *A. flavus* | NP |  |  |  |  |  | 0.25 | 0.25 | 0.5 | 2 | ++ | + |
| LMC9011.01 | *A. flavus* | NP |  |  |  |  |  | 0.25 | 0.5 | 0.5 | 2 | ++ | +++ |
| LMC9012.01 | *A. flavus* | NP |  |  |  |  |  | 0.12 | 0.25 | 0.25 | 1 | + | + |
| LMC9013.01 | *A. fumigatus s.s.* | - | - | - | - | - | - | 0.5 | 0.25 | 0.5 | 1 | + | + |
| LMC9014.01 | *A. fumigatus s.s.* | - | - | - | - | - | - | 0.5 | 0.25 | 0.5 | 1 | ++ | + |
| LMC9015.01 | *A. fumigatus s.s.* | - | - | - | N248K | - | - | 1 | 0.5* | 2* | 2 | ++ |  |
| LMC9016.01 | *A. fumigatus s.s.* | - | - | - | N248K | - | - | 0.5 | 0.5* | 0.5 | 2 | ++ |  |
| LMC9017.01 | *A. fumigatus s.s.* | - | - | - | - | - | - | 0.5 | 0.5* | 0.5 | 2 | ++ |  |
| LMC9018.01 | *A. fumigatus s.s.* | - | - | - | - | - | - | 1 | 0.25 | 2* | 2 | ++ |  |
| LMC9019.01 | *A. fumigatus s.s.* | - | - | - | - | - | - | 0.5 | 0.25 | 1 | 2 | ++ | +++ |
| LMC9020.01^§ c^ | *A. fumigatus s.s.* | F46Y | M172V | N248T |  | D255E | E427K | 1 | 0.5* | 2* | 2 | + |  |
| LMC9021.01 | *A. fumigatus s.s.* | - | - | - | - | - | - | 0.5 | 0.25 | 0.5 | 1 | ++ | +++ |
| LMC9022.01 | *A. fumigatus s.s.* | - | - | - | - | - | - | 0.5 | 0.12 | 1 | 1 | ++ |  |
| LMC9023.01^§ c^ | *A. fumigatus s.s.* | F46Y | ~~-~~ | - | - | - | E427K | 0.5 | 0.5* | 0.5 | 2 | ++ |  |
| LMC9024.01 | *A. fumigatus s.s.* | - | - | - | - | - | - | 1 | 0.5* | 1 | 2 | ++ |  |
| ATCC204304 | *A. flavus* | NP |  |  |  |  |  | 0.25 | 0.25 | 0.25 | 2 | ++ | +++ |
| ATCC46645 | *A. fumigatus s.s.* | - | - | - | - | - | - | 0.5 | 0.25 | 0.5 | 4* |  | +++ |
| *, values > ECV (M59 protocol, CLSI and Espinel-Ingroff et al., 2018) with capture of ≥97.5% of the statistically modeled population; ^§^, silent mutations G89G, L358L and C454C; ^c^, -335 bp T→C and -70 bp C→T nucleotides substitutions upstream *cyp51A* coding region; ITR, itraconazole; POS, posaconazole; VOR, voriconazole; AMB, amphotericin B; NP, not performed; +, low; ++, medium; +++, high biofilm producers or virulenve. | | | | | | | | | | | | | |
